# How (long) did you sleep last night?

**DOI:** 10.1093/sleep/zsaf172

**Published:** 2025-06-18

**Authors:** Jari K Gool, Mink S Schinkelshoek, Rolf Fronczek

**Affiliations:** Department of Neurology, Leiden University Medical Centre, Leiden, The Netherlands; Stichting Epilepsie Instellingen Nederland (SEIN), Sleep-Wake Centre, Heemstede, The Netherlands; Anatomy and Neurosciences, Amsterdam UMC Location Vrije Universiteit Amsterdam, Amsterdam, The Netherlands; Department of Neurology, Leiden University Medical Centre, Leiden, The Netherlands; Stichting Epilepsie Instellingen Nederland (SEIN), Sleep-Wake Centre, Heemstede, The Netherlands; Department of Neurology, Leiden University Medical Centre, Leiden, The Netherlands; Stichting Epilepsie Instellingen Nederland (SEIN), Sleep-Wake Centre, Heemstede, The Netherlands

When asked “How did you sleep last night?”, people generally remember how they felt after waking up and respond accordingly. Yet, if one had performed a polysomnography the night before, the relationship between sleep features and the subjective feeling of having slept well is far from so clear. A more difficult question might be “How long did you sleep last night?”. Most people turn out to be able to answer this reasonably well. However, people with chronic insomnia very often *under*estimate how long they slept. This makes sleep state misperception a key feature of the disorder [1]. But what about people who *over*estimate their total sleep duration? Might this be a feature of idiopathic hypersomnia?

It is often challenging to explain idiopathic hypersomnia to people in the consultation room. It is a brain disorder (a “central disorder of hypersomnolence”). Yet, unlike narcolepsy type 1, we do not know the cause. This is why doctors call it “idiopathic”. We rule out other causes and try to objectify either increased need for sleep or increased sleep pressure. Seemingly arbitrary, a cut-off point of 11 hours was diagnostically chosen. If one sleeps more than this during a day, we formally call it a “disorder”. Otherwise, it is still within “normal” limits. Yet, also if you have a normal daily sleep duration but fall asleep within 8 minutes throughout the day (without REM-sleep occurring too often), you have this “disorder” [2]. If that is not complex enough: when diagnostic tests are repeated, the diagnosis may change to narcolepsy type 2, or to “normal” [3]. Recently, the perhaps counterintuitive use of the hypnotic sodium oxybate in idiopathic hypersomnia turned out to reduce excessive daytime sleepiness in a striking way [4]. It is clear that we do not understand the cause(s) of this disorder at all.

Adam and his colleagues are conducting research at the Sleep and Wake Disorders Centre in Montpellier, which has a long and distinguished history of studying idiopathic hypersomnia [5]. They took an interesting new approach in their paper “Sleep overestimation differentiates between participants with suspected idiopathic hypersomnia” to try to grasp this disorder. Sleep state misperception was studied, more specifically, sleep overestimation. Participants were individuals suspected of having idiopathic hypersomnia and healthy controls. Each underwent a polysomnography, a multiple sleep latency test and a 32-hour bed-rest protocol without any circadian synchronizers. Based on these assessments, participants were diagnosed with either idiopathic hypersomnia (with or without long sleep time) or “non-specified hypersomnia”. The primary outcome measure was the discrepancy between perceived and actual sleep duration during the 32-hour bed-rest protocol. Participants later diagnosed with idiopathic hypersomnia *without* long sleep time or non-specified hypersomnia tended to overestimate their sleep duration, while healthy controls and those with idiopathic hypersomnia *with* long sleep time were generally more accurate in their estimates.

The authors conclude that a subgroup of people with hypersomnolence complaints actually overestimates their sleep duration. Perhaps paradoxically, overestimation of sleep (or underestimation of wake time) occurs in those who don’t objectively have an increased sleep duration. This raises the broader question of whether clinical attention in this population should be more focused on wakefulness rather than on sleep. It is tempting to draw parallels with insomnia, as this disorder is also characterized by subjective and objective sleep quality misalignment [1]. This suggests an opportunity for cognitive behavioral therapy (CBT) in “paradoxical” hypersomnia. CBT for insomnia (CBT-i) has been proven highly effective and certain components may also be beneficial for those with “paradoxical” hypersomnolence complaints. Particularly, cognitive therapy and stimulus control focused on addressing unwanted thoughts on sleep state misperception and sleep–wake patterns would be interesting modalities for future studies.

Meanwhile, it is important to realize that the current diagnosis of idiopathic hypersomnia represents a heterogeneous group of patients, subdivided into normal and long sleep time phenotypes [2]. This “lumping” has hindered research uncovering underlying pathophysiologies [6]. Better grouping based on phenotypes is critical to improve our understanding. Adam and colleagues provide important arguments that further emphasize the need to differentiate long sleepers from those with normal sleep durations using objective measures, such as polyso mnography or actigraphy. The classical cutoff of >11 hours of daily sleep might be reconsidered. More flexible diagnostic criteria for the long sleeper phenotype might be warranted, with inclusion of frequently accompanying markers (such as sleep drunkenness, refreshing nature of naps, and weekend-week sleep length difference) [7]. Also, it would be of particular value to see the current study replicated in a real-world setting.
